# Plasmonic Metalens to Generate an Airy Beam

**DOI:** 10.3390/nano13182576

**Published:** 2023-09-17

**Authors:** Citlalli T. Sosa-Sánchez, Ricardo Téllez-Limón

**Affiliations:** 1 Unidad Foránea Monterrey, Centro de Investigación Científica y de Educación Superior de Ensenada, Alianza Centro 504, PIIT, Apodaca 66629, Mexico; csosa@cicese.mx; 2 CONAHCYT—Unidad Foránea Monterrey, Centro de Investigación Científica y de Educación Superior de Ensenada, Alianza Centro 504, PIIT, Apodaca 66629, Mexico

**Keywords:** metalenses, plasmonics, airy beam, metaphotonics

## Abstract

Airy beams represent an important type of non-diffracting beams—they are the only non-diffracting wave in one dimension, and thus they can be produced with a cylindrical geometry that modifies a wavefront in one dimension. In this paper, we show the design of a cylindrical plasmonic metalens consisting of an array of nanoslits in a gold thin layer that modulates the phase of a Gaussian beam to generate an airy beam propagating in free space. Based on the numerical results, we show that it is possible to generate an airy beam by only matching the phase of wavefronts coming out from the array of gold nanoslits to the airy beam phase at plane z=0. We numerically demonstrate that the airy beam exhibits bending over propagation and self-healing properties. The transmission efficiency is around 60%. The simplicity of the proposed structure open new perspectives in the design of flat metasurfaces for light-focusing applications.

## 1. Introduction

Airy wave packets have been proposed by Berry and Balazs on the quantum mechanical context as free-spreading solutions of the time-independent 1D Schrödinger equation [1], because of the existent isomorphism between this equation and the paraxial Helmholtz equation. They have been studied in different fields such as optics [2,3,4], acoustics [5,6] and plasmonics [7,8,9,10,11,12,13,14]. Airy wave packets constitute a very special class of an accelerating optical wave; in fact, it is the only non-diffracting wave possible in one dimension [2]. So, an airy beam is self-healing, non-diffracting and has the unique property of freely accelerating without the presence of external forces. All these properties make an airy beam attractive for several applications such as optical micro-manipulation [15], laser processing [16] or light-sheet microscopy [17].

Usually, airy beams are generated using spatial light modulators (SLM) [18,19,20,21,22] or by Fourier transforming [7]. Both methods involve the use of bulky optical elements preventing the compactness or portability of the optical systems. To overcome this size limitation, flat metasurfaces have been proposed to generate plasmonic airy beams in metallic layers [23,24,25,26,27,28,29], as well as airy beams in free space. For instance, in [30], an array of subwavelength slits was used to simultaneously generate an airy beam propagating in free-space and an Airy surface plasmon polariton (SPP). In [31], an array of hyperbolic metamaterials was used to generate microwave airy beams. In [32], achromatic terahertz airy beams were generated with an array of micrometer-sized solid and inverse rectangular structures on a silicon substrate. In [33], circular GaN nanopillars were used to design a cubic-phase metasurface, and in [34], an array of meta-atoms was used to generate airy beams. However, the large majority of works using metasurfaces to generate airy beams make use of light scatterers of complex geometries, representing several challenges in terms of fabrication.

Because of the ease of their fabrication, plasmonic metalenses (PM) based on metallic nanoslits have been widely studied [35,36,37,38,39]. This technology utilizes nanoscale structures to manipulate the flow of light, leading to exciting possibilities for new applications such as high-resolution imaging, spectroscopy and as color-routers to guide different light colors into different spatial positions [40,41,42]. The operation principle of a PM is based on the phase matching of light passing through each nanoslit to the phase of a desired wavefront.

In this contribution, we demonstrate the generation of an airy beam propagating in free space through a cylindrical PM consisting of an array of metallic nanoslits of variable width *w* in a thin metallic layer of thickness *t*. The width of each nanoslit was computed in such a way that only the phase of the airy beam can be modulated. We show the bending and self-healing properties of the airy beam, the wavelength dependence of the metalens design and its transmission efficiency.

## 2. Design of the Metalenses

**Figure 1:**
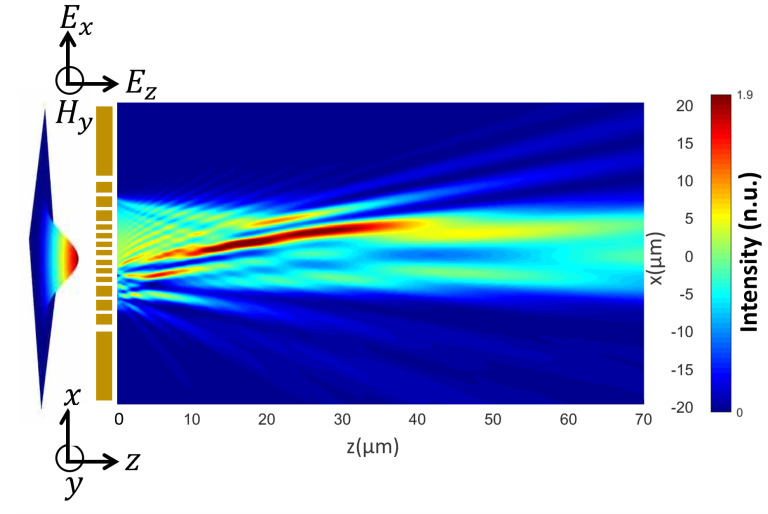
Schematic of airy beam generation with a plasmonic metalens. A p-polarized Gaussian beam illuminates the metasurface (nanoslits array), and the nanoslits are infinite and invariant along y-axis. A proper distribution of the nanoslit widths leads to light structuration and generates an airy beam.The metalens is not shown at true scale.

The PM used to generate the airy beam is an array of metallic nanoslits of width *w* in a gold (Au) film of constant thickness *t*. This array is invariant along the *y* direction, and the nanoslits are distributed along the *x* axis, as shown in Figure 1. The nanoslit width, *w*, is determined by matching the phase profile of the airy beam with the phase shift experienced by the wavefront at the exit of each nanoslit. The airy beam is the only non-diffracting beam in one-dimension, so the symmetry requires phase modulation over only one axis. Hence, by symmetry arguments, the compact and simple PM proposed, which modifies the phase in only one dimension, is suitable to generate the airy beam.

Each nanoslit was modeled as a metal–insulator–metal plasmonic waveguide for *p*-polarized light [43]. The propagation constant of plasmonic guided modes is given by [36,44]:

(1) $$\tanh\left(\frac{w}{2}\sqrt{\beta^{2}-k_{0}^{.}2\varepsilon_{air}}\right)=-\frac{\varepsilon_{air}}{\varepsilon_{m}}\frac{\sqrt{\beta^{2}-k_{0}^{2}\varepsilon_{m}}}{\sqrt{\beta^{2}-k_{0}^{2}\varepsilon_{air}}}$$

where β and $k_{0}$ are the TM mode waveguide and free-space propagation constants, respectively, and $\varepsilon_{air}$ and $\varepsilon_{m}$ are the dielectric constants for air and metal, respectively. When *p*-polarized light impinges the slit, it will excite SPPs propagating with a propagation constant β until they uncouple into light in free space, at the exit of the slit. Light propagated through the nanoslit will have a phase change of $\phi_{PM}=\beta t$ as said before; by matching this phase with the airy phase and solving Equation (1), the corresponding width *w* is obtained. The metalens will require *p*-polarized light to work as shown in Equation (1), and it will be wavelength dependent.

A 1D airy beam is obtained by solving the paraxial wave equation in one dimension:

(2) $$\frac{\partial^{2}\psi }{\partial x^{2}}+2ik_{0}\frac{\partial \psi }{\partial z}=0$$

according to that, the solution is given by the following scalar wave:

(3) $$\begin{array}{cc} \psi \left(x,z\right)=Ai\left[\frac{x}{x_{0}}-{\left(\frac{z}{2k_{0}x_{0}^{2}}\right)}^{2}+i\left(\frac{az}{k_{0}x_{0}^{2}}\right)\right] & \exp\left[i\left(\frac{xz}{2k_{0}x_{0}^{2}}+\frac{a^{2}z}{k_{0}x_{0}^{2}}-\frac{1}{12}{\left(\frac{z}{k_{0}x_{0}^{2}}\right)}^{3}\right)\right]\\ \; & \times \exp\left[a\frac{x}{x_{0}}-\frac{a}{2}{\left(\frac{z}{k_{0}x_{0}^{2}}\right)}^{2}\right] \end{array}$$

Equation (3) represents the propagation of an airy beam with exponential apodization *a*, which should be a positive number and significantly less than one, Ai(x) is the airy function, $x_{0}$ is the bending parameter, $k_{0}$ is the free-space wavevector, and *z* is the propagation direction.

Due to its non-diffracting nature, the airy beam can be generated with the initial field envelope at z=0, which is given by

(4) $$\psi \left(x,z=0\right)=Ai\left(\frac{x}{x_{0}}\right)\exp\left(a\frac{x}{x_{0}}\right)$$

We need to equate the phase change produced by the nanoslit with the phase of an airy beam. For this particular case, the airy beam is generated by matching its initial phase distribution at the metalens plane z=0, which is given by the argument of Equation (4). Thus, the nanoslit phase distribution must satisfy

(5) $$\phi_{PM}=\phi_{Ai}\left[\psi (x,z=0)\right],$$

from equation $\phi_{PM}=\beta t$ and the argument of Equation (4),

(6) $$\beta t=\arg\left[Ai\left(\frac{x}{x_{0}}\right)\exp\left(a\frac{x}{x_{0}}\right)\right]$$

**Figure 2:**
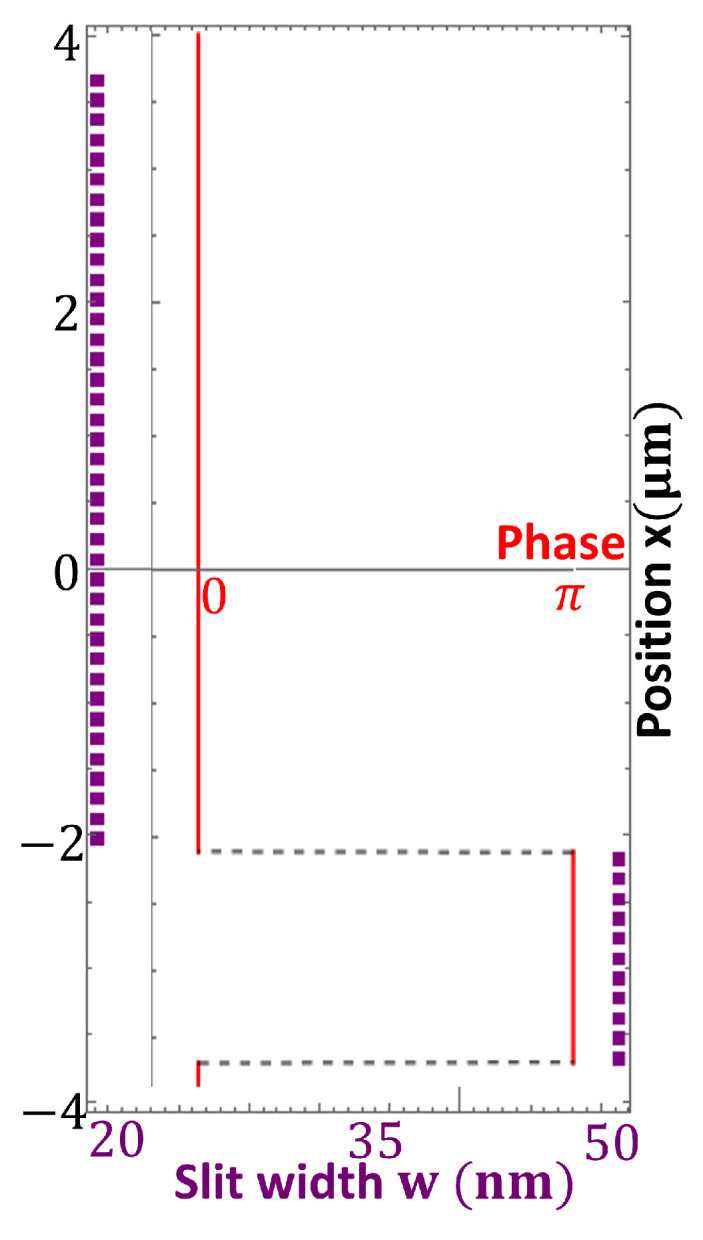
Calculated distribution of the slit width to match the phase of an airy Beam. The red plot shows the behavior of the airy beam phase at plane z=0, which is the plane where the PM was located. The purple squared dots illustratively show the position distribution along the *x*-axis and the widths of every nanoslit necessary to generate the phase of an airy beam, when the PM array is composed of 50 nanoslits. The intensity distribution is scaled to the intensity of the incident beam.

From Equation (6), it can be seen that the phase of an airy beam presents and abrupt change behavior—it jumps from 0 to π as shown in the red plot of Figure 2. According to Equation (1), this implies that only two different widths of the nanoslits are necessary to reproduce the airy beam phase at z=0. The width size strongly depends on the thickness of the metallic film.

The design we proposed was computed with a gold metal film whose thickness was t=210 nm, 101 nanoslits separated by a center to center distance of 150 nm, apodization parameter a=0.04, wavelength λ=784 nm, dielectric constant of air $\varepsilon_{d}=1$, dielectric constant of gold $\epsilon_{gold}=0.14860+i4.7747$, taken from [45], and the bending of the beam was $x_{0}=0.9\;\mu$m. With these parameters, two widths for the nanoslits were obtained, 20 nm and 50 nm (purple square dots in Figure 2), which were achievable values with current nanofabrication techniques. In order to avoid light leakage at the PM ends, two rectangular slits of 30μm each were placed, so the total length of the PM device was 75μm. For this design, we obtained two width values for the nanoslits: $w_{1}=20$ nm and $w_{2}=50$ nm. For the simulations, the waist of the incident Gaussian beam was $GB_{w}=12\;\mu$m, which gives the maximum value of transmission efficiency.

To validate the generation of the airy beam with the designed PM, we performed numerical simulations by means of the integral equation method [46]. This rigorous method solves the Maxwell equations though Green’s second integral theorem. The profile of the PM was discretized and replaced by a collection of punctual light sources, resulting from the interaction of the incident Gaussian beam with the rest of the punctual sources defining the profile. This method allows us to fully determine the transmission efficiency of the system in terms of the angular spectrum. For our PM, we used a separation between adjacent punctual sources of λ/30, where λ corresponds to the wavelength of the incident Gaussian beam. To develop the script, Refs. [46,47,48] were followed. The metalens is invariant along the *y* direction, which means that TM polarization was defined by the electromagnetic field components $\vec{F}=\left(E_{x},H_{y},E_{z}\right)$. This polarization condition allows the generation and propagation of surface plasmons at each nanoslit.

## 3. Results

**Figure 3:**
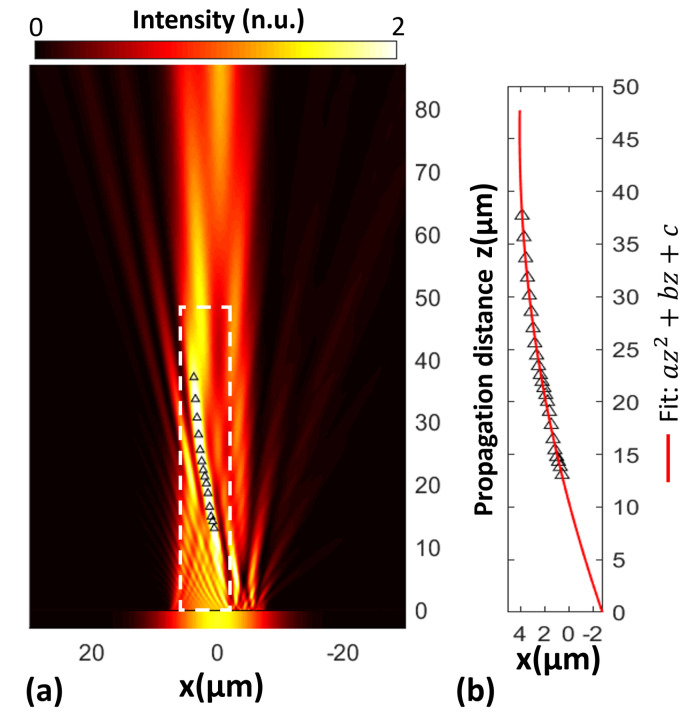
Simulation of an airy beam produced by the PM and its corresponding fit. (**a**) The plot shows the intensity pattern of the transmitted light (propagated through vacuum) that results from the incident gaussian beam on the PM array. The PM is composed of 101 gold nanoslits of thickness t=210 nm, whose widths are $w_{1}=20$ nm and $w_{2}=50$ nm; it is designed for λ=784 nm, $\epsilon_{gold}=0.14860+i4.7747$. The bending parameter of the airy beam is $x_{0}=0.9\;\mu$m, truncation factor a=0.04, and the waist of the incident gaussian beam is $GB_{w}=12\;\mu$m. (**b**) The plot shows the fit associated with the region of maximum intensity, between 0–48μm. This demonstrates the parabolic trajectory of the maximum. Intensity distribution is scaled to the intensity of the incident beam.

The nanoslits array we designed successfully generated the airy beam; indeed, after a long propagation, the parabolic trajectory was preserved. The PM was designed for a wavelength λ=784 nm and 101 nanoslits. Figure 3a shows the map of transmitted light. To verify the parabolic trajectory, the maximum intensity values in the region between 10–38 μm were fitted using the method of non-linear squares. The lowest degree polynomial that best fit the curve was a univariate quadratic function of one-variable (z), $az^{2}+bz+c$. For an example, we show in Figure 3b, a=−0.00325μm, b=0.29780μm, and c=−1μm, and the r-squared value (percent of variance) was $R^{2}=0.9955$. Thus, we can truly say that the trajectory followed by the intensity maximum was a parabola. It is worth mentioning that for a smaller number of nanoslits, the parabolic trajectory is not preserved.

**Figure 4:**
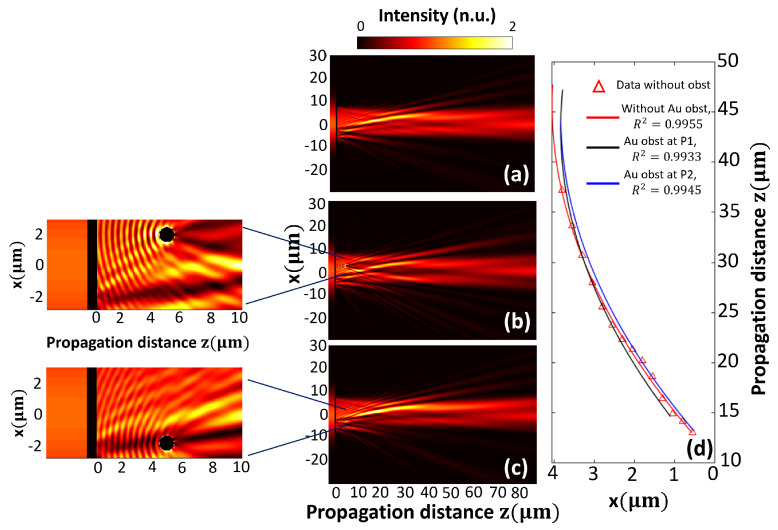
Airy beam self-healing comparison for different obstacle positions. (**a**) PM without obstacle, (**b**) with Au circular obstacle (r=0.5μm) placed at $P_{1}=\left(x,z\right)=\left(2\;\mu m,5\;\mu m\right)$, and (**c**) with the same Au circular obstacle placed at $P_{2}=\left(-2\;\mu m,5\;\mu m\right)$. (**d**) The plot shows three different fits for the cases: (1) red curve, when there is no obstacle, (2) vlack curve, when there is an Au circular obstacle at P1 and (3) blue curve, Au circular obstacle at P2. Intensity distribution is scaled to intensity of the incident beam.

To show the change in behavior when there was an obstacle placed in front of PM, several simulations were conducted. The self-healing property of the airy beam was proven by placing a circular Au particle of radius r=0.5 μm, at positions P1=(2μm,5μm) and P2=(−2μm,5μm). The intensity map in Figure 4a corresponds to the case without an obstacle. When the obstacle was placed at position P1 (Figure 4b), it can be noted that the obstacle caused a perturbation in the near region, but after some microns (approximately at 10μm), the behavior of the airy beam was recovered. When the obstacle was placed at position P2 (Figure 4c), the intensity map is barely modified. In Figure 4d, the comparison between three fits for the region of maximum intensity is shown. The curves described the deflection over *x* axis as the beam was propagating. The r-squared values are shown in Figure 4d inset, and the corresponding values of the quadratic and linear parameter of every fit were (a=−0.00325,b=0.29780), (a=−0.00331,b=0.28748) and (a=−0.00336,b=0.30001) for the red (without obstacle), black (with an obstacle placed at P1) and blue curve (with an obstacle placed at P2), respectively. Again, the lowest degree polynomial that best fit the curves was a univariate quadratic function of one-variable (z), $az^{2}+b^{z}+c$.

**Figure 5:**
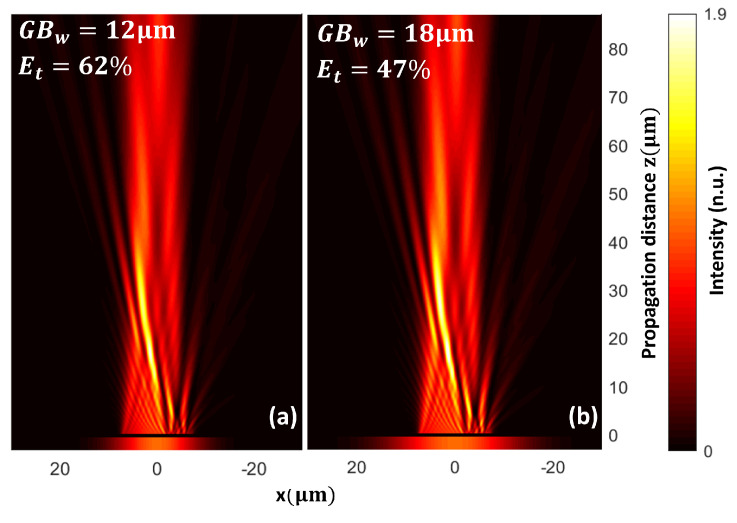
Comparison of transmission efficiency for different waist values of the incident Gaussian beam on PM. (**a**) Intensity map when the PM is illuminated with a Gaussian beam of waist $GB_{w}=12\;\mu$m, and the transmission efficiency is 62%. (**b**) Intensity map when the PM is illuminated with a Gaussian beam of waist $GB_{w}=18\;\mu$m, and the transmission efficiency is 47%.

We also compared the transmission efficiency ($E_{T}$) of the PM as a function of the waist of the incident Gaussian beam. For instance, for a waist $GB_{w}=12$ μm (Figure 5a), the transmission efficiency was about 62%, and the power absorbed by the PM was 3.9%. For a waist $GB_{w}=18$ μm (Figure 5b), the transmission efficiency was about 47%, and the power absorbed by the PM is 5%. This difference arises from the fact that the effective length covered by the PM is 15μm; a Gaussian beam smaller than the PM implies less energy loss, while a bigger waist of the incident Gaussian beam implies that not all the energy is crossing the PM plane, diminishing its efficiency.

**Figure 6:**
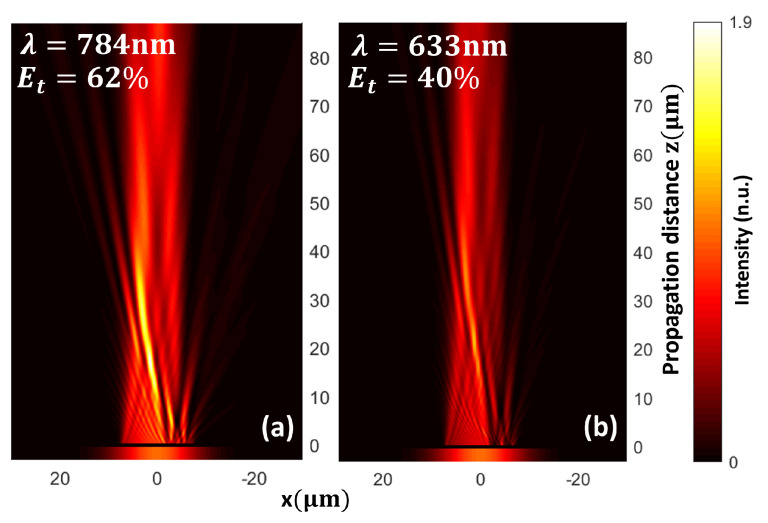
Chromatic dependence of the PM for airy beam generation. Intensity maps for PM designed for an operating wavelength λ=784 nm when illuminated with a Gaussian beam at (**a**) λ=784 nm with transmission efficiency $E_{T}=62\%$ and (**b**) λ=633 nm with transmission efficiency $E_{T}=40\%$.

Furthermore, to evaluate the chromatic dependence of the design, we showed that for a PM designed to operate at a wavelength λ=784 nm (Figure 6a), when it was illuminated with $\lambda^{\prime }=633$ nm (Figure 6b), a lower contrast airy beam was generated. The Figure 6b was normalized to the maximum intensity value of Figure 6a.

## 4. Conclusions

Based on the numerical results, we show that it is possible to generate an airy beam by only matching the phase of wavefronts coming out from the array of gold nanoslits to the airy beam phase at plane z=0. We have shown that a line of nanoslits can be used to generate an airy beam without modulating light amplitude. There are many parameters involved in the design of PM: the metal dielectric constant, thickness and width of the slit, waist and wavelength of the incident Gaussian beam, bending parameter $x_{0}$ and truncation factor *a* of the exponential apodization. The width of the nanoslits strongly depends on the choice of material and its thickness. We analyzed the chromatic dependence of the PM. Even when the incident wavelength was modified, the airy beam was still generated, only being affected by the transmission efficiency and contrast of the beam. The transmission efficiency of PM for λ=784 nm was 62%. and the power absorbed by the PM was 3.9%. The results obtained demonstrate that a simpler PM is sufficient to have the desired bending trajectory of the airy beam. The self-healing property of the airy beam was proven by showing that after placing an Au obstacle (circular particle of radius r=0.5μm) at different positions, the parabolic trajectory followed by the main lobe is recovered.

## Data Availability

The data presented in this study are available on request to corresponding author.
